# High Throughput Approaches to Unravel the Mechanism of Action of a New Vanadium-Based Compound against *Trypanosoma cruzi*

**DOI:** 10.1155/2020/1634270

**Published:** 2020-04-11

**Authors:** M. Florencia Mosquillo, Pablo Smircich, Analía Lima, Sergio A. Gehrke, Gonzalo Scalese, Ignacio Machado, Dinorah Gambino, Beatriz Garat, Leticia Pérez-Díaz

**Affiliations:** ^1^Laboratorio de Interacciones Moleculares, Facultad de Ciencias, Universidad de la República, Montevideo, Uruguay; ^2^Departamento de Genómica, Instituto de Investigaciones Biológicas Clemente Estable, Montevideo, Uruguay; ^3^Unidad de Bioquímica y Proteómica Analíticas, Institut Pasteur de Montevideo, Montevideo, Uruguay; ^4^Biotecnos, Technology and Science, Montevideo, Uruguay; ^5^Department of Biotechnology, Catholic University of Murcia, Murcia, Spain; ^6^Área Química Inorgánica, Facultad de Química, Universidad de la República, Montevideo, Uruguay; ^7^Área Química Analítica, Facultad de Química, Universidad de la República, Montevideo, Uruguay

## Abstract

Treatment for Chagas disease, a parasitosis caused by *Trypanosoma cruzi*, has always been based on two drugs, nifurtimox and benznidazole, despite the toxic side effects described after prolonged prescription. In this work, we study a new prospective antitrypanosomal drug based on vanadium, here named V^IV^O(5Brsal)(aminophen). We found a good IC_50_ value, (3.76 ± 0.08) *μ*M, on CL Brener epimastigotes. The analysis of cell death mechanism allowed us to rule out the implication of a mechanism based on early apoptosis or necrosis. Recovery assays revealed a trypanostatic effect, accompanied by cell shape and motility alterations. An uptake mostly associated with the insoluble fraction of the parasites was deduced through vanadium determinations. Concordantly, no drastic changes of the parasite transcriptome were detected after 6 h of treatment. Instead, proteomic analysis uncovered the modulation of proteins involved in different processes such as energy and redox metabolism, transport systems, detoxifying pathways, ribosomal protein synthesis, and proteasome protein degradation. Overall, the results here presented lead us to propose that V^IV^O(5Brsal)(aminophen) exerts a trypanostatic effect on *T. cruzi* affecting parasite insoluble proteins.

## 1. Introduction

Chagas disease is caused by the protozoan parasite *Trypanosoma cruzi* that is mainly transmitted to the mammalian host by blood-sucking triatomine bugs. It can also be congenitally transmitted, from infected mother to child, through blood transfusion and organ transplantation or by ingestion of contaminated food. Currently, there are around 8 million infected people, 10,000 annual deaths, and approximately 25 million people living in risk zones, mainly rural regions of Latin America [1]. Chagas disease remains the most important parasitic disease in this region and is recognized by the World Health Organization as one of the 20 Neglected Tropical Diseases. Although Chagas disease is endemic in Latin America, it has been getting increased attention due to its dissemination to nonendemic countries (USA, Canada, Spain, Australia, and Japan, among others) [2]. The emigration from Latin America of unknowingly infected people and the lack of controls of blood transfusion and organ transplants may have constituted the reason for the disease spreading.

Current chemotherapy is based on two almost 50-year-old drugs: benznidazole and nifurtimox. Both show severe side effects, controversial efficacy in chronic phase, and drug resistance development in some regions. Therefore, new less toxic and more effective drugs are needed. Although many natural and synthetic compounds have been assayed for activity against *T. cruzi*, only a few of them have advanced to clinical trials but were unsuccessful [1, 3, 4]. In the last decades, the inorganic medicinal chemistry field has demonstrated its capacity to develop prospective metal-based drugs for the treatment of parasitic diseases [5–11]. Since then, vanadium has emerged as a metal with remarkable potential for medical uses in insulin-enhancing and antitumor compounds [12–14]. Recently, some efforts have been dedicated to the development of vanadium prospective agents against neglected parasitic diseases [7, 15].

**Figure 1:**
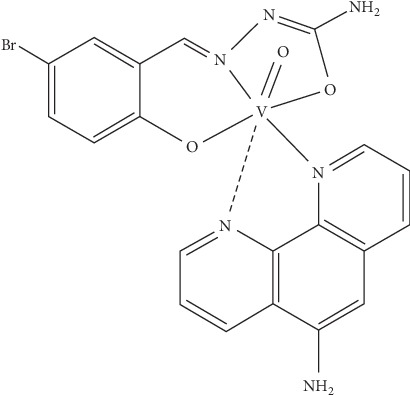
Molecular formula of V^IV^O(5Brsal)(aminophen).

Looking for activity against *T. cruzi*, our group designed oxidovanadium (IV)-based compounds including polypyridyl ligands (NN) with DNA intercalating capacity [16–23]. In particular, a family of 37 structurally related [V^IV^O(L-2H)(NN)] complexes, including semicarbazones of salicylaldehyde derivatives as coligands *L*, has turned out to be promising based on its submicromolar half-maximal inhibitory concentration (IC_50_) range and high selectivity in comparison to mammalian cells. Among them, the 5-bromosalicylaldehyde semicarbazone derivative, [V^IV^O(L-2H)(NN)], where *L* is 5-bromosalicylaldehyde semicarbazone and NN is 5-amino-1,10-phenanthroline, here named V^IV^O(5Brsal)(aminophen) for simplicity (Figure 1), stood out, showing an IC_50_ value of 0.27 *μ*M on *T. cruzi* (Tulahuen 2 strain epimastigotes) and a selectivity of 185 using J774 macrophages [20].

The stability in solution of V^IV^O(5Brsal)(aminophen) towards solvolysis and/or oxidation was previously studied by electron paramagnetic resonance (EPR) and V-51 nuclear magnetic resonance (NMR) [20]. Only a partial oxidation leading to [V^V^O_2_(5Brsal-2H)(solvent)], after displacement of the aminophen heteroligand, was observed. This new V(V) species was demonstrated to be nonactive on *T. cruzi*, and free aminophen showed a twenty-time higher IC_50_ value on the parasite than the original V^IV^O(5Brsal)(aminophen) species [19, 20]. In addition, V^IV^O(5Brsal)(aminophen) showed a 97-fold higher selectivity than aminophen [20]. Therefore, the intact initial V^IV^O(5Brsal)(aminophen) compound was considered as the active species.

Here we studied the mode of action of this vanadium compound as a prospective agent against *T. cruzi* (CL Brener strain). We analyzed the cell death mechanism involved and parasite recovery response. In addition, the amount of the vanadium uptaken by the parasite and its association with parasite macromolecules were determined. Finally, proteomics and transcriptomics strategies were undertaken to identify putative pathways or possible molecular targets affected. To our knowledge, this is the first *omics* study of these characteristics performed on a metal-based prospective agent against *T. cruzi*.

## 2. Materials and Methods

### 2.1. Reagents and V^IV^O(5Brsal)(Aminophen) Synthesis

The 5-bromosalicylaldehyde semicarbazone ligand was synthesized and characterized as previously reported from an equimolar mixture of 5-bromosalicylaldehyde and semicarbazide [24]. For the synthesis of the [V^IV^O(L-2H)(NN)] complex, a suspension in ethanol, previously purged with nitrogen, 0.375 mmol of *L*, 0.375 mmol of 5-amine-1,10-phenanthroline, and 0.375 mmol of [V^IV^O(acac)_2_], where acac denotes acetylacetonate, was heated at reflux under nitrogen for 4 h, and the reddish brown solid formed was filtered off, washed with ethanol and diethyl ether, and characterized by C, H, and N elemental analyses and by Fourier-transform infrared (FTIR) and EPR spectroscopies as previously reported [20, 22].

### 2.2. Parasites and Cell Culture

*T. cruzi* epimastigotes (CL Brener strain) were maintained at 28°C in the Brain Heart Infusion (BHI) medium supplemented with 10% fetal bovine serum and passed every three days.

### 2.3. Determination of *In Vitro* Anti-*Trypanosoma cruzi* Activity

Anti-*T. cruzi* activity was determined following a previously reported method [25–27]. Briefly, an 11.25 mM V^IV^O(5Brsal)(aminophen) solution was prepared in dimethyl sulfoxide (DMSO). Epimastigotes were counted using the Neubauer chamber, and 1 × 10^6^ parasites/mL were incubated in a 96-well plate in 200 *μ*L BHI containing up to 16 *μ*M V^IV^O(5Brsal)(aminophen). Initial parasite density at time 0 (*A*_0_) and parasite proliferation after five days of incubation (*A*_5_) were determined by absorbance at 595 nm. Inhibition of proliferation was calculated considering the proliferation of control parasites incubated with the DMSO vehicle alone, by the following equation: %parasite proliferation=(*A*_5_−*A*_0_)/(A_5c_−A_0c_) × 0100, where A_5c_ and A_0c_ correspond to the optic density of control cultures on day 5 and 0, respectively. In all cases, DMSO never exceeded a final concentration of 0.04%. A dose-response curve was constructed and the IC_50_ value was determined using GraphPad Prism version 6.00 for Windows (GraphPad Software). The results are presented as averages ± SD (standard deviation) of six independent biological replicates.

### 2.4. Cell Death Mechanism

Cell death mechanism analysis was performed using the Dead Cell Apoptosis Kit (Thermo Fisher Scientific). Parasites were incubated for 24 h with 1x, 5x, and 10x IC_50_ of V^IV^O(5Brsal)(aminophen), harvested by centrifugation, washed with phosphate-buffered saline (PBS), and incubated for 15 min with 5 mg/mL Alexa Fluor® 488 annexin V (AV) and 10 mg/mL propidium iodide (PI), followed by dual-parameter analysis using an Accuri C6 (BD Bioscience) flow cytometer. For AV and PI detection, a 533/30 nm signal detector (FL1) and a 670 nm long pass signal detector (FL3) were used, respectively. Two independent experiments were performed in duplicate for each V^IV^O(5Brsal)(aminophen) concentration, and 10,000 events were acquired in each one. Data were analyzed using BD CSampler software (BD Bioscience). Nontreated parasites were used as a control. For apoptosis and necrosis positive control, parasites were treated for 2.5 h with H_2_O_2_ 50 *μ*M and 100 *μ*M, respectively.

### 2.5. Live/Dead Assay

Cell viability was assessed with Calcein AM (CA) and propidium iodide (PI) (Thermo Fisher Scientific). Nontreated parasites and compound-treated parasites were harvested by centrifugation after 24 h of incubation and resuspended in 0.1 mL 1x PBS containing 0.1 mM of CA and 10 mg/mL of PI. Samples were incubated for 60 min at RT and immediately analyzed by flow cytometry with a 533/30 nm filter (FL1) for CA and a 670 nm long pass filter (FL3) for PI. The fluorescence intensity of two independent experiments was acquired for 10,000 events, and the data were analyzed using BD CSampler software (BD Bioscience).

### 2.6. Morphology Analysis

For scanning electron microscopy, 1 × 10^6^ parasites in exponential growth phase, untreated or treated for 6 h with 5x IC_50_ of V^IV^O(5Brsal)(aminophen), were washed with PBS and fixed with 4% paraformaldehyde. The samples were then subjected to dehydration with increasing concentration of acetone (30–100%) and gold-coated in the Metallizer Sputter Coater SCD050/LEICA. After metallization, samples were observed in a scanning electron microscope (Philips XL30, Eindhoven).

### 2.7. Recovery Assays

Recovery assays of *T. cruzi* epimastigotes were performed as previously described [25, 28]. Epimastigotes were incubated for 4 h with 1x, 5x, and 10x IC_50_ of V^IV^O(5Brsal)(aminophen) and washed with and transferred to fresh compound-free BHI. Parasite proliferation was followed at 595 nm in a Thermo Scientific Varioskan® Flash Multimode for 24, 48, and 72 h. To calculate relative proliferation, untreated control parasites were used.

### 2.8. V^IV^O(5Brsal)(Aminophen) Uptake Determination and Macromolecule Association Analysis

Vanadium uptake was determined by incubating the parasites with V^IV^O(5Brsal)(aminophen) followed by electrothermal atomic absorption spectrometry in a Thermo iCE 3500 spectrophotometer (Thermo Fisher Scientific). Epimastigotes (1 × 10^7^ parasites/mL) were incubated for 24 h with 1, 5, and 10x IC_50_ of the vanadium compound. At the indicated time points, 8 × 10^7^ parasites were collected by centrifugation, washed once, and resuspended in PBS for vanadium quantification. Noninternalized vanadium in the supernatant was also determined. Two independent experiments were performed for each of the three concentrations evaluated.

To determine the association of vanadium with nucleic acids (3 × 10^7^ parasites), Wizard®GenomicDNAPurificationKit(Promega) and TRIzol Reagent (Life Technologies) for DNA and RNA isolation, respectively, were used. For protein analyses, parasites (4 × 10^7^) were resuspended in 1 mL of Parasite Lysis Buffer containing 10 mM Tris-Cl pH 7.5, 1 mM EDTA, 1% CHAPS, 10% glycerol, 0.5% Triton, and Complete™ProteaseInhibitorCocktail(Roche); stirred 30 min at 4°C; and centrifuged at 4°C for 1 h at 20,000g to separate soluble from insoluble fraction. The associated vanadium was then determined in each fraction. Two independent experiments were performed for all analytical determinations and for each sample, and two vanadium determinations were performed in each one.

### 2.9. Transcriptome and Proteomic Analysis

Total RNA was isolated from parasites (1 × 10^9^), untreated and treated with 5x IC_50_ V^IV^O(5Brsal)(aminophen) during 6 h, using TRIzol (Life Technologies) reagent following the manufacturer's instructions (three independent replicas for each one). PoliA + RNA pair-end sequencing was performed at Macrogen using Illumina TruSeq™ RNA Sample Preparation Kit v2 and HiSeq 2500 (http://www.macrogen.com). Trimmomatic [29] was used to obtain good quality sequence reads that were mapped to the *T. cruzi* genome (version 29, http://tritrypdb.org) using Bowtie 2 in very sensitive mode [30]. The number of sequence reads per gene was determined using htseq-count [31]. Differentially expressed genes were determined using the DESeq2 package [32].

Total proteins from parasites (1 × 10^9^), untreated and treated with 5x IC_50_ V^IV^O(5Brsal)(aminophen) during 6 h, were isolated by incubation with lysis buffer (7 M urea, 2 M thiourea, 4% CHAPS) for 30 min at 4°C, centrifuged at 4°C for 1 h at 20,000g to separate soluble from insoluble fractions, and analyzed by LC tandem-mass spectrometry (LC-MS/MS) (three independent replicas). Briefly, 20 *μ*g of each sample was resolved in precast 4%–12% gradient gels (NuPAGE, MES System, Invitrogen). Each lane was cut and processed for mass spectrometry analysis according to previously reported protocols [33]. Peptide samples were fractionated into a nano-HPLC system (EASY-nLC 1000, Thermo Scientific) equipped with a reverse-phase column (PepMap™, RSLC, C18, 2 m, 100 Å, 50 mm × 15 cm, Thermo Scientific) using a gradient from 50% to 100% of 0.1% formic acid in acetonitrile at a flow rate of 250 nL/min. Peptide analysis was performed in a LTQ Velos nano-ESI linear ion trap instrument (Thermo Scientific) operated in data dependent acquisition mode (top 10) with a dynamic exclusion list of 45 s. Data analysis was carried out using PatternLab for Proteomics software (version 4.0.0.84). Raw files were searched against a target-decoy database generated from *Trypanosoma cruzi* (strain CL Brener) sequences downloaded from UniProt (http://www.uniprot.org), applying the following parameters: trypsin as proteolytic enzyme with full specificity, methionine oxidation as variable modification, cysteine carbamidomethylation as fixed modification, and 800 ppm of tolerance from the measured precursor *m/z.* PatternLab's Approximately Area Proportional Venn Diagram and T-Fold modules were used to quantitatively analyze data [34–36].

### 2.10. Real-Time PCR

Quantitative RT-PCR (qRT-PCR) using SensiFAST SYBR Hi-ROX Kit (Bioline) was performed on 10 ng/*μ*L of cDNA obtained from RNA of each transcriptome biological replica using random primers and Superscript II (Life Technologies). Specific primers were designed with the OligoPerfect Primer Designer tool from Thermo Fisher Scientific (https://www.thermofisher.com). The threonyl-tRNA synthetase gene was used as an internal amplification control, since its transcript level does not present variations between treated and control parasites according to the obtained transcriptomic data. The reactions were carried out containing the primers in a final concentration of 0.4 *μ*M with an annealing temperature of 60°C in a StepOnePlus Real-Time PCR System. All reactions were performed in duplicate. The data obtained were processed with StepOnePlus™ software v2.3, and the determination of the relative values of RNA was performed by the 2^−ΔΔCT^ method [37].

## 3. Results

### 3.1. Growth Inhibition Mechanisms of V^IV^O(5Brsal)(Aminophen) in T. *cruzi* Epimastigotes

**Figure 2:**
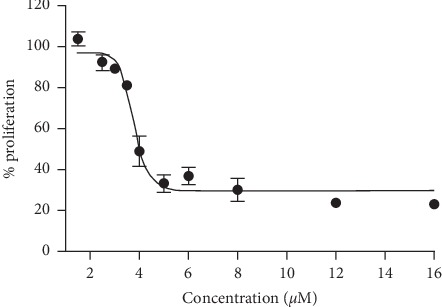
Effect of V^IV^O(5Brsal)(aminophen) on T. *cruzi* epimastigotes proliferation.

As a first step to study the antiproliferative mechanism of action of V^IV^O(5Brsal)(aminophen) on *T. cruzi* epimastigotes, we analyzed the growth inhibition curve and determined the IC_50_, (3.76 ± 0.08) *μ*M, in CL Brener strain (Figure 2).

Parasites were grown at 28°C for 5 days in BHI with the indicated concentrations of V^IV^O(5Brsal)(aminophen). Each point represents the mean of six independent experiments with the associated standard error.

**Figure 3:**
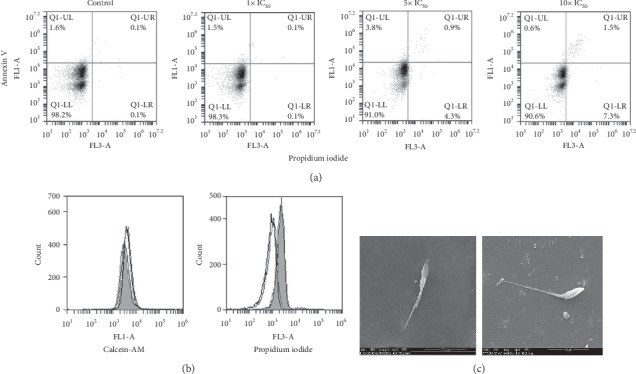
Cell death mechanism and morphological analysis of V^IV^O(5Brsal)(aminophen) treated T. *cruzi* epimastigotes. (a) Flow cytometry analysis of parasites, untreated (control) and treated with V^IV^O(5Brsal)(aminophen) for 24 h at the indicated concentrations (1x IC_50_, 5x IC_50_, and 10x IC_50_), labeled with annexin V and propidium iodide. Dot plots represent unlabeled parasites (lower left square), early apoptotic annexin V labeled parasites (upper left square), and late apoptotic/necrotic annexin V/propidium iodide double labeled parasites (upper right square). (b) Flow cytometry analysis of parasites, untreated (black line) and incubated with the vanadium-based compound for 24 h at 1x IC_50_ (gray line), 5x IC_50_ (solid filled black line), and 10x IC_50_ (solid filled gray line), labeled with Calcein AM (left panel) and with propidium iodide (right panel). (c) Scanning electron microscopy image of a representative control untreated parasite (left image) and epimastigotes treated with 5x IC_50_ of V^IV^O(5Brsal)(aminophen) for 6 h. Images were obtained with a Philips XL30 scanning electron microscope (magnification of 10,000 x.)

To find out if cell death mechanisms such as early apoptosis or necrosis/late apoptosis were involved, we performed flow cytometry analysis with the fluorescent hallmark probes AV and PI as described previously [25–27]. The absence of AV and/or PI positive cells after incubation with either 1x, 5x, or 10x IC_50_ for 24 h suggests that neither apoptosis nor necrosis processes were triggered by the V^IV^O(5Brsal)(aminophen) treatment (Figure 3(a)). Similar results were obtained for 6 and 12 h of incubation. To determine whether treated parasites were dead or growth arrested, a live/dead assay was performed using fluorescent Calcein AM. As shown in Figure 3(b), the cell esterase activity was not affected (with 1x IC_50_) or slightly decreased (with 5x and 10x IC_50_). In addition, no drastic morphological changes in VIVO(5Brsal)(aminophen) treated parasites could be observed by scanning electron microscopy when using 5x IC_50_ (Figure 3(c)). However, a tendency to have shortened cell bodies and a clear loss of motility (not shown) was observed.

**Figure 4:**
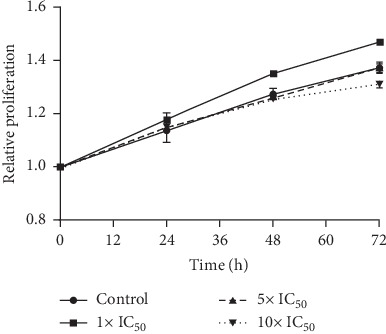
Recovery experiment of T. *cruzi* epimastigotes treated with V^IV^O(5Brsal)(aminophen). The growth of parasites exposed for 4 h to 0, 1x, 5x, and 10x IC_50_ V^IV^O(5Brsal)(aminophen) after compound removal was followed by optic density measures (595 nm) at the indicated time points. Proliferation was determined relative to time zero. Each experiment was performed in triplicate, and the mean and standard deviation were represented for each point.

Considering that these results prove that classical cell death mechanisms are not involved in the parasite growth inhibition of V^IV^O(5Brsal)(aminophen), we investigated the existence of a trypanostatic effect through the recovery assay described by Kessler [28]. After an incubation period, enough to induce cell death mechanisms, similar parasite growth recovery profiles were observed for untreated and treated parasites at all the concentrations tested (Figure 4). This result confirms a trypanostatic mechanism of action for the V^IV^O(5Brsal)(aminophen) since the parasite's growth is inhibited in presence of the compound, but parasites can resume growth in its absence.

### 3.2. Vanadium Uptake in *T. cruzi* Epimastigotes Treated with V^IV^O(5Brsal)(Aminophen)

**Figure 5:**
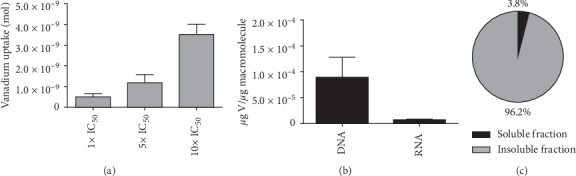
Uptake and macromolecule association of V^IV^O(5Brsal)(aminophen) in T. *cruzi* epimastigotes. (a) Amount of vanadium strongly bound and/or uptaken by compound-treated parasites. Parasites were incubated with 3.76 *μ*M (1x IC_50_), 11.28 *μ*M (5x IC_50_), and 37.6 *μ*M (10x IC_50_) of V^IV^O(5Brsal)(aminophen), and incorporated vanadium was determined through electrothermal atomic absorption spectrometry. (b) Percentage of total vanadium associated with DNA, RNA. (c) Determination of total vanadium associated with soluble and insoluble fraction. Vanadium amounts were determined through electrothermal atomic absorption spectrometry after 6 h of incubation with 5x IC_50_. The average of two independent experiments is presented with its associated standard deviation.

In order to advance towards understanding the molecular mechanism of the trypanostatic action, we estimated the amount of vanadium uptaken by the parasites. As shown in Figure 5(a), parasites exhibit a dose dependent vanadium uptake with values of 0.50, 1.18, and 3.51 nmol for 1x, 5x, and 10x IC_50_ of V^IV^O(5Brsal)(aminophen), respectively. It is worth noting that the parasite uptake only reaches on average the 2.4% of the vanadium present in the incubation mixture (total vanadium: 15 nmol for 1x IC_50_). This low uptake hampers the analysis of a detailed subcellular distribution of the vanadium associated with the parasites.

Considering the capacity of the ligand to intercalate within nucleic acids [19], the distinctive association with the parasite DNA and RNA was analyzed. Despite the low values of vanadium associated with the parasite nucleic acids (8.9 × 10^–5^ *µ*g V/*µ*g DNA and 6.46 × 10^–6^ *µ*g V/*µ*g RNA), a favored association with DNA was observed (Figure 5(b)).

Nonetheless, using a simple fractionation approach, a preferred association of the metal with the insoluble fraction (more than 90% of the uptaken vanadium) was observed (Figure 5(c)). This result suggests that V^IV^O(5Brsal)(aminophen) could be interacting with membrane associated proteins.

### 3.3. Early Omic Response of *T. cruzi* Epimastigotes to V^IV^O(5Brsal)(Aminophen) Treatment

To identify key molecules as well as possible pathways involved in the mode of action of the V^IV^O(5Brsal)(aminophen), we studied the main omic changes induced in parasites by the incubation with 5x IC_50_ V^IV^O(5Brsal)(aminophen) for 6 h. We selected a relative short time of incubation to study only the early response triggered specifically by the compound, thus avoiding the incidence of general late or/and indirect mechanisms.

For the analysis of transcriptomic changes, three independent biological replicates were sequenced for each condition, and at least 7 million paired-end sequence reads were mapped to the reference *T. cruzi* genome using Bowtie 2 (Table S1). A very good correlation was observed for the replicates (Pearson correlation coefficient *p* > 0.99). The comparative transcriptomic analysis revealed minimal changes on mRNA steady state levels upon treatment. Indeed, from a total of 10,951 mapped transcripts, less than 100 transcripts (0.008%) were differentially expressed, considering a false discovery rate of 0.01. As expected, most of them (47%) codify for hypothetical proteins with no known function or functional information or domains.

Furthermore, only 2 differentially expressed transcripts (encoding for hypothetical proteins) showed a fold change greater than 1.5 (Table S2). qRT-PCR was performed on some genes to confirm transcriptomic data (Figure S1). An excellent correlation between both approaches (transcriptomic and qRT-PCR) for the selected set of genes (R Pearson 0.97) was obtained. For the analysis of proteomic changes, a shotgun strategy was assayed to separately identify the soluble and insoluble protein changes due to the vanadium compound incubation.

The proteomic analysis of insoluble protein fraction revealed a total of 248 overrepresented and 110 underrepresented insoluble proteins in the V^IV^O(5Brsal)(aminophen) parasites compared with control untreated parasites (Table S3). Cytochrome P450 (TcCLB.506945.190) appears to be the most downregulated insoluble protein. Interestingly this protein has been involved in mediating detoxification processes (Table S3). Among the top overrepresented insoluble proteins, we found two acetyltransferases which constitute component of the pyruvate dehydrogenase complex (TcCLB.509717.20, TcCLB.510105.170) and a Glutathione-S-transferase/glutaredoxin (TcCLB.506443.70). Some reductases (TcCLB.506821.210, TcCLB.510819.4), oxidoreductases (TcCLB.507049.60, TcCLB.506485.80), and hydrolases (TcCLB.503399.20, TcCLB.506931.10, TcCLB.506747.30) also appeared overrepresented in the insoluble protein fraction of treated parasites as well as several ribosomal proteins (Table S3).

**Figure 6:**
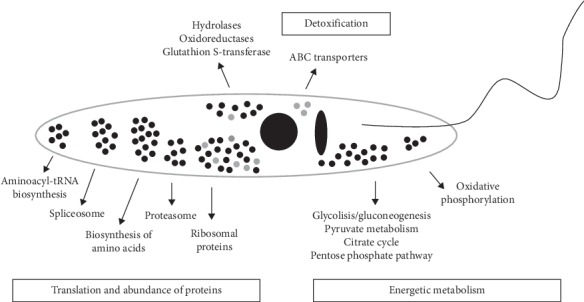
Schematic representation of protein abundance changes in (T). *cruzi* epimastigotes treated with V^IV^O(5Brsal)(aminophen). Proteins are grouped based on KEGG pathways distribution. Black and gray dots indicate proteins that appear overrepresented and underrepresented in V^IV^O(5Brsal)(aminophen) treated vs. untreated parasites, respectively.

Regarding the soluble proteins, 122 appeared underrepresented with 105 of them only present in the untreated controls. Remarkably we found some ATP binding cassette transporters (ABC transporters: TcCLB.506925.530, TcCLB.510943.80, TcCLB.507099.80), with the latter two involved in drug expulsion mechanism [38]. Besides, the abundance of some other proteins related to detoxification processes was also modified (Figure 6). In addition, KEGG pathway analysis revealed that the V^IV^O(5Brsal)(aminophen) treatment affects regulatory processes of protein expression, since the abundance of proteins involved in spliceosome, biosynthesis of amino acids (Gly, Ser, Thr, and His), aminoacyl-tRNA biosynthesis, ribosomes, and proteasome appears to be altered (Table S4). It is interesting to mention that proteasome proteins were also overrepresented among the insoluble proteins (Table S3). Finally, energetic metabolism seems to be also affected by V^IV^O(5Brsal)(aminophen) treatment, as the abundance of many proteins related to carbon metabolism processes, such as citrate cycle, glycolysis, gluconeogenesis, pentose phosphate pathway, pyruvate metabolism, and oxidative phosphorylation, is modified (Table S4).

## 4. Discussion

The aim of this work was to evaluate the cellular and molecular mechanism of action of a new vanadium-based compound in *T. cruzi.* Proliferation assays revealed an IC_50_ value of (3.76 ± 0.08) *μ*M, similar to the one of the reference drug nifurtimox, (2.8 ± 0.2) μM [25, 26]. Thus, V^IV^O(5Brsal)(aminophen) can be visualized as a promising antiproliferative compound.

*T. cruzi.* It is worth noting that complete inhibition is not achieved with the assayed compound concentrations (Figure 2). To understand this issue, we decided to investigate the mode of action of this compound more deeply.

Regarding the mechanism of inhibition of parasite growth, negative AV and PI labeling revealed that neither apoptosis nor necrosis is involved. Moreover, the slight decrease of the metabolic activity, deduced from the esterase analysis, as well as the absence of the characteristic drastic morphological changes, such as cell membrane rupture or cell body increase [38], suggests a growth arrest phenomenon rather than the involvement of another cell death mechanism. Conversely, a trypanostatic mode of action of V^IV^O(5Brsal)(aminophen) on *T. cruzi* was confirmed by recovery assays.

Though the percentage of the compound uptaken by the parasites (less than 2.4% of the total vanadium used for incubation) is similar to that obtained for other well-known metallic antiproliferative compounds such as cisplatin (3%), oxaliplatin (1%), or pyrodach-2 (0.1%) in three different human cell lines [39], it is notably lower than the ones obtained for other metal-based compounds (more than 20%) in *T. cruzi* epimastigotes [25, 26]. Nonetheless, the low V^IV^O(5Brsal)(aminophen) uptake is enough to exert the antiparasitic effect. Although this vanadium-based compound series has been designed including an intercalating molecule, and its *in vitro* DNA association has been reported [19], in the current *in vivo* assays only vanadium traces associated with nucleic acids could be detected. Anyway, this low amount could be enough to affect DNA replication accounting for the cytostatic effect observed in the recovery assays. On the other hand, a striking association with the insoluble fraction (90%) was found. It is worth mentioning that this fraction mainly contains membrane associated proteins but also includes lipids and diverse functional groups in posttranslationally modified proteins.

High throughput analyses have been only recently implemented to analyze the mechanisms involved in kinetoplastid drug resistance and the mode of action of drugs [40, 41]. Aiming to deepen the study of the molecular mechanism of action of V^IV^O(5Brsal)(aminophen) on *T. cruzi*, we undertook the omics analysis of early altered transcripts and proteins because of the treatment of parasites with this novel compound. Transcriptomic analysis revealed a few differentially expressed transcripts (less than 100, being less than 1%) with low levels of up- or downregulation (most of them have log_2_ Fold change < 0.5), which may be in accordance with the low *in vivo* association of vanadium with the parasite nucleic acids. Nonetheless, the importance of these changes must not be underestimated. For example, a very tiny modification in the expression of a given regulatory protein could result in a global effect in the parasite if it participates in key metabolic pathways. The low effect of vanadium compound on the parasite transcriptome can be explained by its preferred *in vivo* association with the insoluble fraction rather than with nucleic acids (Figure 5). On the other hand, we found that 14% of the total identified proteins (523 out of 3,638) were differentially abundant after treatment, most of them in the insoluble protein fraction (68%) (Figure S2). The number of 3,638 total proteins detected is in good agreement with previous *T. cruzi* proteomics analysis, which has reported the identification of 2,784 [42] and 4,920 proteins [43]. Proteomics has only been employed in Chagas disease chemotherapy studies to analyze the mode of action of naphthoimidazoles with 30 differentially abundant proteins detected in epimastigotes [44] and 61 in trypomastigotes [45]. Conversely, our results reflect a pleiotropic molecular effect of V^IV^O(5Brsal)(aminophen). Many of the differentially expressed proteins in the insoluble fraction have been reported to be involved in mediating detoxification processes. Among them Cytochrome P450 is worth noting, whose diminished abundance in treated parasites may explain the lack of an effective detoxification response [46]. Other interesting proteins that appear differentially expressed include acetyltransferases, reductases, and hydrolases. While the involvement of transferases, oxidases, reductases, or hydrolases in the modification of internalized compound has been reported [47, 48], these results suggest a major role of V^IV^O(5Brsal)(aminophen) driving to early energy and redox metabolic disorders. Since many ribosomal proteins were differentially expressed in treated parasites (Table S3), despite the minimal association of the compound with mRNA (Figure 5), we can speculate that the translation process could be affected. Among the diminished soluble proteins, we found transporters of the ABC family whose role in drug expulsion mechanisms has been previously proposed [49]. In this way, the compound elimination could be reduced, thus allowing prolonging its trypanostatic effect. Besides, in our proteomic analysis, proteasome proteins appear overrepresented in treated parasites in both soluble and insoluble protein fractions. The proteasome has recently been studied as the target of promising drugs for malaria, leishmaniasis, Chagas disease, and sleeping sickness [50, 51].

It is tempting to speculate that the possible direct inhibition of proteasome by V^IV^O(5Brsal)(aminophen) could lead to an increase in proteasome protein production to hamper the compound effect. The overrepresented proteasome proteins could be also explained by the existence of extensive protein modifications requiring a more active proteasome to degrade them.

Altogether, the data here presented support the idea of a main mode of action of V^IV^O(5Brsal)(aminophen) through the modification of the abundance of numerous parasite proteins. While the altered protein content driven by the V^IV^O(5Brsal)(aminophen) may be due to its direct association with the protein targets promoting their degradation or stabilization, the compound may be also specifically affecting proteins involved in the protein production and degradation processes, thus expanding the effect.

## 5. Conclusion

In conclusion, this work provides a thorough description of the cellular and molecular responses of *T*. *cruzi* epimastigotes to a prospective new vanadium-based antiparasitic compound. Interestingly, the very good IC_50_ value determined for V^IV^O(5Brsal)(aminophen) is reached through a trypanostatic mode of action, moderately affecting cell shape and motility. The parasite early response to this compound displays no drastic changes in mRNA levels. Nonetheless, proteomic analysis revealed a wide effect on protein abundance identifying specific proteins potentially involved in this massive response and in the drug metabolism. The combined use of omic approaches allowed us to identify the affected processes that drive a very good inhibition of parasite growth even at the very low uptake levels detected. Globally, the results here presented constitute a contribution to understand the mechanism of action of this potential drug for the treatment of Chagas disease. Furthermore, they contribute an insight into metallomics, proteomics, and transcriptomics of metal-based antitrypanosomal compounds, encouraging this incipient research area.

## Data Availability

The transcriptomic and proteomic data used to support the findings of this study are included within the supplementary information files; raw data can also be obtained by emailing the corresponding author.

## Supplementary Materials

Supplementary Materials Figure S1: validation of transcriptomic data of selected genes with qRT-PCR. The expression of random selected modified transcripts from transcriptome data list was analyzed by qRT-PCR comparing untreated and treated parasites, and expression values (log_2_ FC RT-PCR) were plotted against transcriptomic data (log_2_ FC transcriptomic). Figure S2: determination of differentially abundant proteins in *T. cruzi* epimastigotes treated with V^IV^O(5Brsal)(aminophen). Soluble (A) and insoluble (B) proteins from untreated (control) and treated parasites were analyzed. The figure shows a Volcano plot generated with the T-Fold module from PatternLab for Proteomics. Proteins detected in at least 4 replicates of all conditions were indicated as individual dots and plotted accordingly to the *p *value (log_2_ (p value)) and fold change (log_2_ (fold change)). Black dots represent proteins that do not satisfy neither the fold change nor the statistics criteria for differential expression and thus are considered unchanged between conditions. Dark gray represents proteins that do satisfy the fold change but not the statistic criteria for differential expression. Light gray dots represent low abundant proteins satisfying both fold change and q value criteria for differential expression but not considered for further analysis due to the low number of spectral counts. Finally, white dots correspond to proteins satisfying all statistical filters and represent the differentially expressed proteins between strains. For details about proteins corresponding to white dots, see supplemental Table S3. Table S1: row data stats from transcriptome analysis of control untreated parasites and V^IV^O(5Brsal)(aminophen) treated parasites. Table S2: list of differentially expressed genes from transcriptome analysis. The upregulated and downregulated transcripts in V^IV^O(5Brsal)(aminophen) treated parasites with respect to control untreated parasites are shown. Table S3: list of differentially expressed proteins from proteomic analysis. The upregulated and downregulated soluble and insoluble proteins in V^IV^O(5Brsal)(aminophen) treated parasites with respect to control untreated parasites are shown in different tabs. Table S4: KEGG enrichment analysis of metabolic pathways using the STRING database for modified proteins in V^IV^O(5Brsal)(aminophen) treated parasites.
