# Supplementary figures and images for: High Throughput Approaches to Unravel the Mechanism of Action of a New Vanadium-Based Compound against Trypanosoma cruzi
Source: Bioinorg Chem Appl. 2020 Apr 11;2020:1634270. doi: 10.1155/2020/1634270 (PMC7171612; doi:10.1155/2020/1634270)

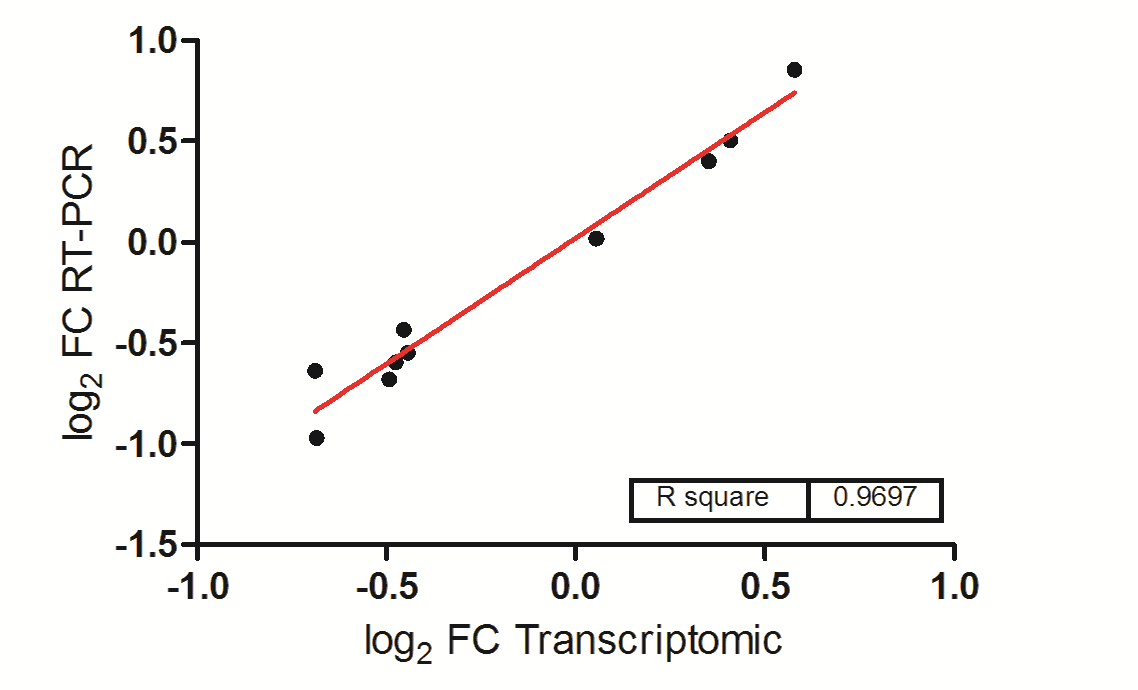

Supplement: Supplementary Materials — Figure S1: validation of transcriptomic data of selected genes with qRT-PCR. The expression of random selected modified transcripts from transcriptome data list was analyzed by qRT-PCR comparing untreated and treated parasites, and expression values (log2 FC RT-PCR) were plotted against transcriptomic data (log2 FC transcriptomic). Figure S2: determination of differentially abundant proteins in T. cruzi epimastigotes treated with VIVO(5Brsal)(aminophen). Soluble (A) and insoluble (B) proteins from untreated (control) and treated parasites were analyzed. The figure shows a Volcano plot generated with the T-Fold module from PatternLab for Proteomics. Proteins detected in at least 4 replicates of all conditions were indicated as individual dots and plotted accordingly to the p value (log2 (p value)) and fold change (log2 (fold change)). Black dots represent proteins that do not satisfy neither the fold change nor the statistics criteria for differential expression and thus are considered unchanged between conditions. Dark gray represents proteins that do satisfy the fold change but not the statistic criteria for differential expression. Light gray dots represent low abundant proteins satisfying both fold change and q value criteria for differential expression but not considered for further analysis due to the low number of spectral counts. Finally, white dots correspond to proteins satisfying all statistical filters and represent the differentially expressed proteins between strains. For details about proteins corresponding to white dots, see supplemental Table S3. Table S1: row data stats from transcriptome analysis of control untreated parasites and VIVO(5Brsal)(aminophen) treated parasites. Table S2: list of differentially expressed genes from transcriptome analysis. The upregulated and downregulated transcripts in VIVO(5Brsal)(aminophen) treated parasites with respect to control untreated parasites are shown. Table S3: list of differentially expressed proteins from [file 1634270.f1.zip › 1634270.f1/S1_Fig.tif]

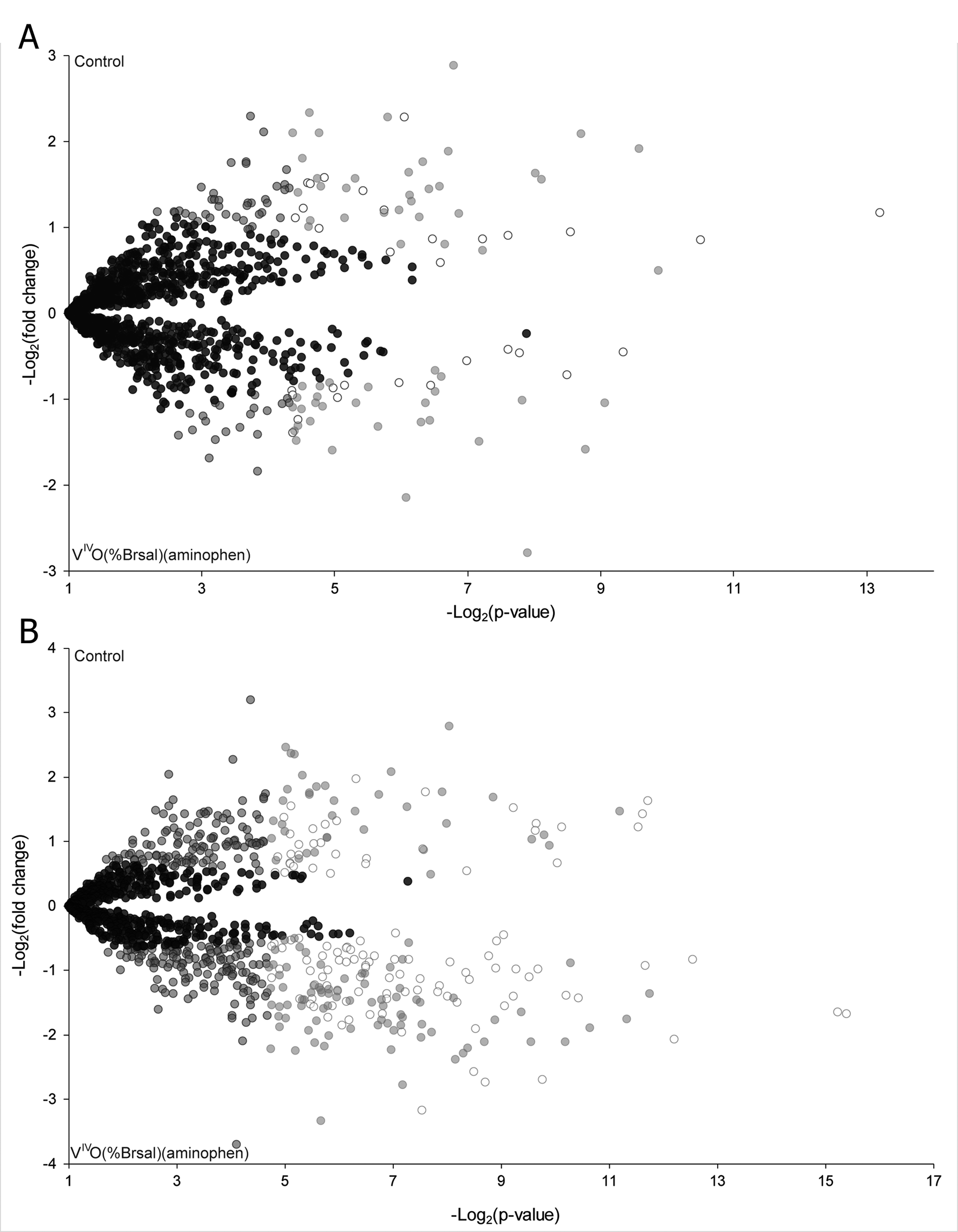

Supplement: Supplementary Materials — Figure S1: validation of transcriptomic data of selected genes with qRT-PCR. The expression of random selected modified transcripts from transcriptome data list was analyzed by qRT-PCR comparing untreated and treated parasites, and expression values (log2 FC RT-PCR) were plotted against transcriptomic data (log2 FC transcriptomic). Figure S2: determination of differentially abundant proteins in T. cruzi epimastigotes treated with VIVO(5Brsal)(aminophen). Soluble (A) and insoluble (B) proteins from untreated (control) and treated parasites were analyzed. The figure shows a Volcano plot generated with the T-Fold module from PatternLab for Proteomics. Proteins detected in at least 4 replicates of all conditions were indicated as individual dots and plotted accordingly to the p value (log2 (p value)) and fold change (log2 (fold change)). Black dots represent proteins that do not satisfy neither the fold change nor the statistics criteria for differential expression and thus are considered unchanged between conditions. Dark gray represents proteins that do satisfy the fold change but not the statistic criteria for differential expression. Light gray dots represent low abundant proteins satisfying both fold change and q value criteria for differential expression but not considered for further analysis due to the low number of spectral counts. Finally, white dots correspond to proteins satisfying all statistical filters and represent the differentially expressed proteins between strains. For details about proteins corresponding to white dots, see supplemental Table S3. Table S1: row data stats from transcriptome analysis of control untreated parasites and VIVO(5Brsal)(aminophen) treated parasites. Table S2: list of differentially expressed genes from transcriptome analysis. The upregulated and downregulated transcripts in VIVO(5Brsal)(aminophen) treated parasites with respect to control untreated parasites are shown. Table S3: list of differentially expressed proteins from [file 1634270.f1.zip › 1634270.f1/S2_Fig.tif]
